# Knowledge and attitude of traumatic dental injuries in Mongolian schoolteachers

**DOI:** 10.1186/s12903-024-04526-w

**Published:** 2024-07-10

**Authors:** Tsetsegkhen Narantsetseg, Ochbayar Naran-Ochir, Enkhtsatsral Ganbold, Ganbaatar Yunden, Batzorig Bayartsogt, Batbayar Badral, Munkhdul Altannamar, Enkh-Orchlon Batbayar

**Affiliations:** 1 Department of Oral and Maxillofacial Surgery, School of Dentistry, Mongolian National University of Medical Sciences, Zorig street, P.O.Box-48/111, Ulaanbaatar, 14210 Mongolia; 2 Megadent Oral Care Center, Zaisan street, Ulaanbaatar, 17013 Mongolia; 3 Department of Epidemiology and Biostatistics, School of Public Health, Mongolian National University of Medical Sciences, Zorig street, Ulaanbaatar, 14210 Mongolia; 4 Department of Oral and Maxillofacial Surgery, School of Dentistry, Ach Medical University, Peace Avenue, Ulaanbaatar, 18101 Mongolia

**Keywords:** Dental trauma, Traumatic dental injuries, Knowledge, Teachers, Child

## Abstract

**Background:**

Traumatic dental injury (TDI) is a growing public health concern worldwide, and children and adolescents are commonly affected. Because TDI often occurs at school, the response of teachers to these injuries is crucial. However, teachers in various countries have been shown to lack knowledge of effective TDI first-aid response and need an intervention to improve their knowledge. The aim of the study presented here was to ascertain and analyze teachers’ knowledge of and attitude about TDI in Mongolia.

**Materials and methods:**

A cross-sectional study of full-time teachers in Mongolia was performed using an online questionnaire (compiled from relevant studies) from September 2022 to December 2022. The questionnaire consists of 47 items and among them 14 were used to assess the teacher’s knowledge, and 5 were for attitude towards TDI. The maximum possible score was 14 points and grouped as follows good, moderate, and poor. T-test, ANOVA test (post-hoc) and linear regression analysis were performed.

**Results:**

The online survey provided quantitative data from 2821 participants: 28% were elementary school teachers, 29% middle school teachers, and 42% high school teachers. Their mean age was 36.7 ± 9 years, and 2433 (86%) were female. The mean score of the TDI knowledge of the teachers was 5.3 ± 2.1 out of 14. The ANOVA test showed that older (*p* < 0.01) and more experienced teachers (*p* < 0.01) had higher scores. Teachers in the eastern (5.46 ± 2.2) and southern (5.49 ± 2.2) provinces had higher mean scores than in the other provinces (*p* < 0.02). Teachers majored in natural sciences (5.4 ± 2.2) had a higher score than those in the social sciences (5.2 ± 2.1) and difference were significant (*p* < 0.02). The multiple regression model statistically significantly predicted a one-year increase in experience, the TDI score increased 0.034 (95% CI 0.026, 0.043) unit.

**Conclusions:**

The knowledge regarding TDI and effective first-aid response to such injury is low in teachers in Mongolia. However, the teachers’ attitude about TDI was positive and they wanted to learn and improve their knowledge. Therefore, further education and training programs are advised.

## Introduction

Traumatic dental injury (TDI) is one of the biggest public health problems around the world; more than one billion people – mainly children [1–4]. – have experienced TDI [1]. The injuries have various causes, such as falls, accidents and collisions, sports and recreational activities, and physical alterations. The study of Pacheco found that around 22–30% of children experienced TDI while attending school [5, 6]. Recent studies have shown that TDI in children may occur in school environments due to falls or during playing or fighting. Educational institutions are considered high-risk settings for TDI [7–10]. According to a meta-analysis of world TDI prevalence, 15.2% of TDI cases involved damage to permanent dentition, and 22.7% involved damage to primary dentition [1]. Furthermore, domestic violence was an essential etiologic factor of TDI. The aggression happened in the form of punches and slaps against the child, and the most frequently injured teeth were the incisors [11]. 

Therefore, as potential first responders to traumatic incidents, the teachers’ knowledge regarding TDI is crucial to providing effective first aid to the victims [12–15]. Timely first aid benefits the victims in many ways, including survival of traumatized teeth and diminishing trauma-related stress [16, 17]. Previous studies have also shown that delayed first aid or inadequate treatment can lead to functional, financial, and psychological issues in victims of TDI [10, 18]. Unfortunately, a number of studies have also shown that teachers worldwide lack adequate knowledge of TDI [3, 12, 14, 15, 19–22]. The Ivanda study conducted a questionnaire-based study to assess teachers’ knowledge about TDI and included 803 full-time teachers. Their knowledge main score was 6.6 ± 2.5 out of 14, and the study concluded that teachers’ knowledge was poor [15]. Studies of Tzimpoulas show the importance of teachers’ experience in the case of TDI, and teachers with more than ten years of experience scored significantly higher than less experienced teachers [20]. 

According to a recent systematic review, the awareness level of TDI was unknown in several areas of the world, including northern parts of Asia. The review recommended that studies be conducted in the regions that lack this data [3]. To our knowledge, no studies have been found on TDI in Mongolia and teachers’ knowledge about TDI. Mongolia is a vast and sparsely populated country, with approximately two persons per square kilometer. Half of the population lives in the capital, and the other half is scattered throughout the country. Primary school starts at 6 years old in Mongolia, and enrollment is compulsory. Due to their nomadic lifestyle, some herders are required to send their children to boarding schools during the school year [23, 24]. As a result, the teachers are the main caregivers at such schools. Moreover, the main disadvantage of previous studies is the lack of a well-designed questionnaire to assess the teacher’s knowledge and attitude [3]. Therefore, careful questionnaire development is necessary.

The aim of the study presented here was to assessing teachers’ level of knowledge about TDI and identifying the factors associated with this knowledge.

## Method

This study was approved by the Ethics Committee of Mongolian National University of Medical Sciences (2022/3–03) and was performed in accordance with the ethical principles stated in the Declaration of Helsinki. Participation was voluntary and the answers to the questionnaire were processed anonymously. The participants gave their informed consent automatically when they began the online questionnaire. Participants were allowed to complete the questionnaire only once.

The cross-sectional study was conducted between September 2022 and December 2022. Inclusion criteria were full-time teachers in Mongolia from elementary school (1st -4th grade), middle school (5th -8th grade), and high school (9th -12th grade), with at least one year teaching experience.

The questionnaire was based on articles from the relevant journals [12, 15, 21, 22]. Three authors (Ts.N, E.O.B, M.A) independently reviewed previous articles and proposed the content and format of the questions. After several rounds of discussions all three agreed on the final English version of the questionnaire. The questionnaire consisted of objective-type questions (multiple choice) It included demographic questions (age, sex, location, experience of TDI at school, school type: governmental or private school etc.); knowledge assessment questions (avulsion, crown fracture, subluxation, recognizing deciduous; and permanent tooth), and attitude questions attitude towards TDI.

The final English version of questionnaire was translated to Mongolian by two authors independently. After reaching consensus, the final Mongolian version of the questionnaire was back-translated by an English translator blinded for the original English version [25]. Lastly, a pilot study was conducted with 20 teachers (not included in the data analysis) in-person. The participants of the pilot study reported no difficulties reading and understanding the questionnaire, and Cronbach’s alpha was 0.967.

The link to the online questionnaire was distributed to the teachers’ email address with support of of General Education Center, Ministry of Education, Mongolia. To assess knowledge about TDI (first aid for avulsion, crown fracture, subluxtion, and regonizing decisous and permamant tooth, handling and transporting injured tooth), correct answers were scored as 1, and incorrect answers were scored as 0 [15]. Single choice questions and multiple-choice questions were both scored in the same way. The maximum possible score was 14 points. The target population consisted of actively working teachers from elementary schools, middle schools, and high schools in Mongolia. Age, years of experience, and the mean score of TDI were numerical variables, and other variables were classified as categorical variables.

Data was analyzed using Statistical Package for the Social Science version 29 (SPSS, IBM). Results were expressed as frequencies and percentages for dichotomous data and as a mean and standard deviation (SD) for continuous data. Normal distribution was tested using Q-Q plot and histogram. T-test (dependent variable was the mean of knowledge scores, independent variables sex, teaching subjects, personally experienced TDI at school, school type) and ANOVA test (dependent variable was the mean of knowledge scores, independent variables include age groups, years of experience groups, teaching grade, academic degree, number of students teaching per day, location, and own assessment of knowledge of TDI) analysis were used to analyze differences between the groups. Independent variables with *p* < 0.20 in the T-test and ANOVA test were included in the multiple linear regression analysis. Multiple linear regression (adjusted) analysis was used to assess a relationship between the dependent variable (knowledge score) and selected independent variables (age, teaching experience, subjects taught, school location, and the knowledge self-assessment).

The level of significance was set at *p* < 0.05 for all statistical tests.

## Results

A convenience sampling resulted of a total of 2821 teachers from elementary, middle, and high schools in Mongolia participated in this study (Table 1). In total 810 (28%) were teachers in elementary school, 813 (29%) were teachers in middle schools, and 1198 (42%) were teachers in high schools. The mean age of the teachers was 36.7 ± 9 years, and 2433 (86%) were female.

**Table 1:** General characteristics of participants of study

Characteristics		*n*= 2821	%	TDI score (mean ± SD)	*P* value
Gender	Female	2433	86	5.3 ± 2.1	0.183*
	Male	388	14	5.1 ± 2.3	
Age	20–29	675	24	4.9 ± 2.0	0.001**
	30–39	1114	39	5.2 ± 2.1	
	40–49	769	27	5.5 ± 2.1	
	50–59	240	9	5.8 ± 2.1	
	60 and above	23	1	6.2 ± 1.7	
Teaching experience in years	1–5	751	26	4.9 ± 2.0	0.001**
	6–10	529	19	5.1 ± 2.1	
	11–15	507	18	5.3 ± 2.2	
	16 and more	1034	36	5.6 ± 2.1	
Teaching grade	Elementary (1–4)	810	28	5.3 ± 2.1	0.995**
	Middle (5–8)	813	29	5.3 ± 2.2	
	High (9–12)	1198	42	5.3 ± 2.1	
Teaching subject	Natural sciences	1528	54	5.4 ± 2.2	0.022*
	Social sciences	1293	46	5.2 ± 2.1	
Academic degree	Bachelor	2001	70	5.2 ± 2.1	0.160**
	Masters	816	29	5.4 ± 2.9	
	Ph.D.	4	1	4.0 ± 2.1	
Personally experienced TDI at school	Yes	944	34	5.3 ± 2.1	0.192*
	No	1877	66	5.2 ± 2.2	
Number of student teaching (per day)	1–20	192	7	5.3 ± 2.0	0.282**
	21–50	852	30	5.3 ± 2.1	
	51–100	718	25	5.2 ± 2.0	
	101–150	581	20	5.4 ± 2.2	
	151–200	478	17	5.1 ± 2.2	
School type	Government	2769	98	5.3 ± 2.1	0.950*
	Private	52	2	5.8 ± 2.2	
School location	Capital city	645	23	5.2 ± 2.1	0.028**
	Western	663	24	5.1 ± 2.1	
	Eastern	452	16	5.4 ± 2.2	
	North / Khangai	598	21	5.3 ± 2.1	
	South/ Center	463	16	5.4 ± 2.2	
Own assessment of TDI knowledge	Poor	607	21	5.0 ± 2.1	0.001**
	Average	1714	60	5.3 ± 2.1	
	Good	57	2	5.4 ± 2.3	
	Excellent	432	16	5.7 ± 2.1	

*T-test

**ANOVA test

A higher level of knowledge about TDI was observed among more experienced (*p* < 0.01) and older (*p* < 0.01) teachers (Table 1). There was no difference between men and women (*p* > 0.05). Teachers in the eastern (5.46 ± 2.2) and southern (5.49 ± 2.2) provinces had higher mean scores than in the other provinces (*p* < 0.02).

**Fig. 1:**
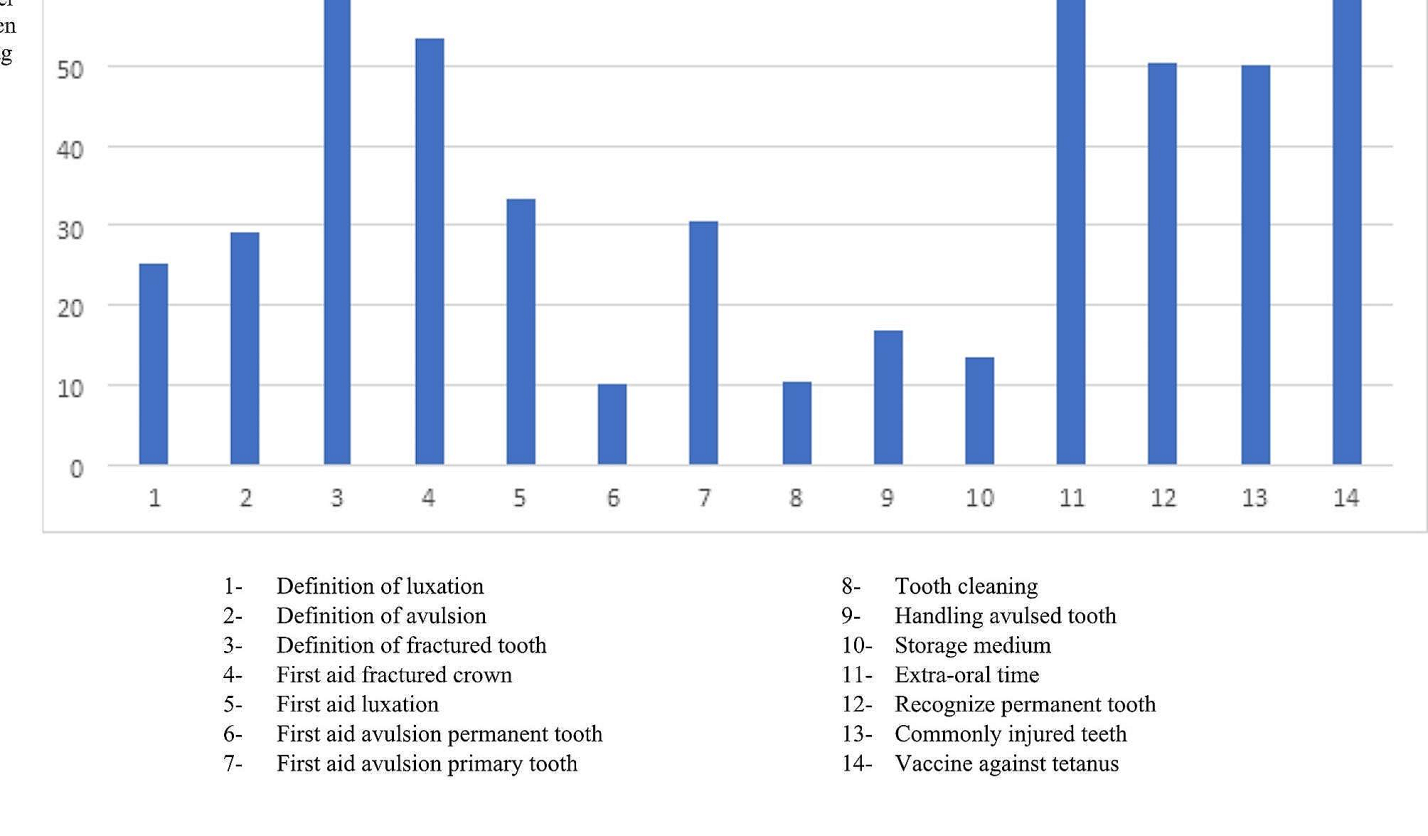
Distribution of accurately answered questions

A higher knowledge score of TDI was found among the teachers who self-assessed their knowledge as sufficient (5.46 ± 2.3) or more than sufficient (5.73 ± 2.1) than those with lower self-assessments. Furthermore, 722 (27%) of the participants reported that they did not have medical professionals at their schools. Teachers who had dentists (5.56 ± 1.9) at their schools had statistically significant (*p* < 0.01) higher mean scores than those who had medical professionals other than dentists (5.49 ± 2.1) at their schools or no medical professionals (4.81 ± 2.2). Among the questions, vaccine against tetanus, extra, -oral time, and fracture tooth definition are the most frequent correctly answered questions whereas management of avulsion permanent tooth, tooth cleaning, and storage media were the least frequent correctly answered (Fig. 1) [15].

In total, 944 (34%) teachers reported that they witnessed TDI in children in the school environment. The characteristics of teachers who witnessed TDI at school are summarized in Table 2. The most common reason for accidents in the school setting was a fall (24%) followed by playing (24%). These accidents happened most at playgrounds (36%) and in classrooms (32%). The teachers’ attitude was positive (Table 3) and half of the participants were willing to participate in the course to improve their knowledge regarding TDI.

**Table 2:** General characteristics of schoolteacher’ witnessed TDI at school

Characteristics		*n*= 944	%
Gender	Female	780	83
	Male	164	17
Age	20–29	237	25
	30–39	381	40
	40–49	238	25
	50–59	80	9
	60 and above	8	1
Teaching experience in years	1–5	258	27
	6–10	177	18
	11–15	168	17
	16 and above	341	36
Teaching grade	Elementary (1–4)	287	31
	Middle (5–8)	249	26
	High (9–12)	408	43
Teaching subject	Natural sciences	527	56
	Social sciences	417	44
Academic degree	Bachelor	680	72
	Masters	263	27
	Ph.D.	1	1
Curriculum	Yes	182	20
	No	785	80
Where happened	Classroom	306	32
	Gym	175	19
	Corridor	121	13
	Playground	342	36
How happened	Running	64	7
	Playing	187	19
	Fall	230	25
	Violence	46	5
	Others	417	44
Which tooth was it	Maxillary central	404	43
	Maxillary lateral	165	17
	Mandibular central	168	18
	Mandibular lateral	207	22
How many teeth	1–2	507	54
	3–4	166	18
	5–10	42	5
	10	31	3
	Cannot remember	198	20
When last time	Within 1 year	364	39
	1–2 years ago,	193	20
	2–5 years ago,	143	15
	5- more years ago	139	14
	Cannot remember	105	12

**Table 3:** Schoolteacher’s attitude regarding TDI

	Agree (%)	Not agree (%)	Do not know (%)
Do you think a teacher must pay attention to the TDI prevention	1494 (53%)	176 (6%)	1151 (41%)
Do you think a teacher should have knowledge about TDI management	1565 (55%)	156 (6%)	1100 (39%)
Do you think TDI requires emergency first aid management	1631 (58%)	140 (5%)	1050 (37%)
Do you think TDI management is only responsibility of dentists	701 (25%)	694 (24%)	1426 (51%)
Do you think teachers participating in a course about TDI management could help improve knowledge	1422 (50%)	210 (8%)	1189 (42%)

A one-way ANOVA was conducted to effect of age on TDI score (Table 1). There was a statistically significant difference between age groups (F(4,2816) = 12.7, *p* < 0.001). A Turkey post hoc test revealed that mean knowledge score of TDI was statistically significantly lower in age group 20–29 (4.98 ± 2.0) compared to 40–49 (5.59 ± 2.1, *p* < 0.001), 49–59 (5.84 ± 2.1, *p* < 0.001), and 60< (6.26 ± 1.7, *p* < 0.039). There was no statistically significant difference between the age group 20–29 and 30–39 groups (*p* = 0.199).

Similarly, a one-way ANOVA was conducted to effect of years of experience on TDI score. There was a statistically significant difference between years of experience groups (F(3,2817) = 14.7, *p* < 0.001). A Turkey post hoc test revealed that the mean knowledge score of TDI was statistically significantly lower in the years of experience 1–5 group (4.99 ± 2.0) compared to the 11–15 (5.38 ± 2.2, *p* = 0.009) and 16< (5.63 ± 2.1, *p* < 0.001). There was no statistically significant difference between the years of experience 1–5 and 6–10 groups (*p* = 0.693).

A multiple regression analysis was conducted to predict mean higher TDI knowledge score from the variables age, teaching experience, subjects taught, school location, and the knowledge self-assessment. The assumption of normality was met, as assessed by a Q-Q Plot. The collinearity between two independent variables (age and years of experience; *r* = 0.914) were observed, therefore the age was removed from the linear regression analysis. The multiple regression model statistically significantly predicted a one-year increase in experience; the TDI score increased by 0.034 (95% CI 0.026;0.043) per unit (Table 4).

**Table 4:** Multiple linear regression model (adjusted) for predictors of teachers’ knowledge about TDI

Variables		Knowledge*			
		B	*P*-Value	95% CI for B	
Year of experience		*0.035*	*0.001*	0.026	0.043
Teaching subject		-0.122	0.134	-0.280	0.037
Location	West	0.107	0.366	-0.125	0.339
	East	0.285	0.029	0.029	0.540
	Center	*0.381*	*0.003*	0.126	0.634
	North/Khangai	0.291	0.106	0.054	0.527
Own assessment of TDI	Excellent	0.146	0.612	-0.418	0.709
	Good	*0.437*	*0.001*	0.212	0.662
	Bad	*-0.288*	*0.004*	-0.486	-0.091

*Continues variable (mean score of the knowledge)

## Discussion

The current study found that overall schoolteacher’s knowledge regarding Traumatic Dental Injuries (TDI) is poor. However, the teacher’s knowledge score increased with age and years of experience. The present study has shown that teachers commonly witness TDI while working, and therefore need to have a basic understanding of first-aid response to such traumatic injuries. Incorporating TDI education into teacher training programs or providing workshops on the topic can equip teachers with the necessary knowledge to respond effectively to TDI, which would ultimately enhance the well-being of children under their care [19, 26]. Unfortunately, our study and others have shown that teachers’ knowledge regarding first-aid response to TDI is insufficient around the world [3, 12–15, 19–22, 26]. Moreover, the schoolteachers are non-medical professionals, and their knowledge regarding the TDI is typically limited. Their primary role is to administer basic first aid and contact emergency services. Interestingly, the general dentist’s knowledge and management of TDI needs to be improved worldwide. The knowledge and management of TDI among general dentists is mainly low to moderate (some studies assessed it as good), and it suggests continuing professional training is needed [22, 27–30]. 

This epidemiological study assessed Mongolian teachers’ knowledge and attitude regarding first-aid response to TDI. In a similar study in Croatia [15], the teachers’ mean score was inadequate, which is identical to our study. Both studies indicate a low level of TDI knowledge. Other studies have shown that the teacher’s age is one of the influencing factors to predict better knowledge regarding TDI [15, 31]. In our study, we observed that older teachers tend to have higher mean scores of TDI than younger teachers. However, this association is different from the findings from other studies [13, 19]. 

Another important finding in our study was that the teachers’ experience predicted a higher level of TDI knowledge, which is broadly supported by other studies [13, 15, 19, 31]. In our study, we found that one more year teaching experience increased the mean knowledge score by 0.035 points. The beta coefficients of the mean score seem to have increased much less points over teachers experience, however, the small increase is acceptable considering the overall low mean score of the participants.

A possible explanation for this might be that teachers with more experience are more likely to be exposed to TDI or other trauma incidents at work or they may have better chance to expose TDI information over a year. Additionally, sex was not predictive of higher knowledge scores in our study, which is in accordance with previous studies [3, 15, 20]. However, other studies found that male teachers are likely to have significantly less knowledge than female teachers [19, 32]. 

In contrast to previous studies [14, 19], we found no difference between teachers with bachelor’s and graduate degrees regarding their TDI knowledge score. In our study, however, teachers who majored in and taught natural science had a higher probability of achieving higher TDI knowledge scores than those in the social sciences. A possible explanation is that in Mongolia, physical education, health education, and biology teachers are classified as teachers of natural sciences.

We found that teachers who worked in the central and eastern regions of the country have better knowledge scores than other regions. A previous study [31] suggested that the region could be an influencing factor on the level TDI knowledge of teachers working there due to the regional socio-economic status. However, our study showed that teachers at schools in the central and eastern regions have more years of experience than those in other regions.

Our study also showed that teachers who assessed themselves as having adequate knowledge regarding TDI had significantly higher scores than those who assessed themselves as having inadequate knowledge. This result agrees with the finding of Ivanda [15]. A multiple linear regression analysis showed that teaching experience (years), school location, and teachers’ self-assessment were predictive of higher TDI knowledge scores.

Approximately one-third of the teachers surveyed in our study indicated that they had witnessed TDI at work. This is in accordance with previous studies [12, 13, 19, 20, 33], which reported that between 29% and 49% of the teachers had witnessed TDI in school settings. In our study, teachers reported that TDI mainly occurred due to falls (24%) and while playing (20%), and the maxillary central incisors (45%) were most often affected. These findings are also consistent with previous studies [12, 15]. This could be because children spend most of their day at school; therefore, TDI frequently occur there. Moreover, it can thus be suggested that access to safe playgrounds at school is crucial to prevent TDI in school environments.

Less than one-third of the teachers of the present study understood the term ‘tooth luxation and avulsion’ whereas around 70% of teachers understood the term ‘tooth fracture’. We found the lowest number of correct answers on questions about tooth avulsion and its handling, cleaning, and storage medium. The lack of this knowledge is problematic due to the importance of correct handling and storage of the avulsion tooth to long-term prognosis.

The generalizability of these results is subject to certain limitations. For instance, data was collected online, some answers could be predictable, and participants may consult any material to answer the questionnaire. A key strength of the present study was the participation of approximately 10% of the total teachers actively working in Mongolia.

This study set out to gain a better understanding of the status of teachers’ knowledge and attitude about TDI in Mongolia. The results show that teachers’ knowledge regarding TDI is low, but their attitude is positive, and they are willing to improve their knowledge. We therefore suggest that schools, education authorities and the government should take these results into account by establishing safer playgrounds in school settings and by improving teachers’ knowledge about TDI.

## Data Availability

The data presented in this study are available on request from the corresponding authors.

## Abbreviations

TDI: Traumatic Dental Injury
